# Monolayer TiAlTe_3_: A Perfect Room-Temperature Valleytronic Semiconductor

**DOI:** 10.3390/ma18102396

**Published:** 2025-05-21

**Authors:** Kang Jia, Chang-Wen Zhang, Zi-Ran Wang, Pei-Ji Wang

**Affiliations:** 1 School of Physics and Technology, Institute of Spintronics, University of Jinan, Jinan 250022, China; 2 School of Physics and Physical Engineering, Qufu Normal University, Qufu 273165, China; 3 Key Laboratory of High-Effciency and Clean Mechanical Manufacture of MOE, School of Mechanical Engineering, Shandong University, Jinan 250061, China; 4 Suzhou Research Institute, Shandong University, Suzhou 215009, China

**Keywords:** ferromagnetic, magnetic transition temperature, valley polarization, anomalous valley Hall effect, ferrovalley

## Abstract

Investigating valley-related physics in rare intrinsic ferromagnetic materials with high-temperature stability and viable synthesis methods is of vital importance for advancing fundamental physics and information technology. Through first-principles calculations, we forecast that monolayer TiAlTe_3_ has superb structural stability, a ferromagnetic coupling mechanism deriving from direct-exchange and superexchange interactions, and a high magnetic transition temperature. We observed spontaneous valley polarization of 103 meV in the bottom conduction band when monolayer TiAlTe_3_ is magnetized toward an out-of-plane orientation. Additionally, because of its powerful valley-contrasting Berry curvature, the anomalous valley Hall effect emerges under an in-plane electric field. The cooperation of ferromagnetic coupling, a high magnetic transition temperature, and spontaneous valley polarization makes monolayer TiAlTe_3_ a promising room-temperature ferrovalley material for use in nanoscale spintronics and valleytronics.

## 1. Introduction

The valley degree of freedom serves as a local minimum (maximum) in the conduction (valence) band, and it can be leveraged to realize information encoding, high-efficiency processing, and non-volatile storage [1,2,3,4,5,6,7,8,9]. The absence of space inversion symmetry in two-dimensional (2D) transition metal dichalcogenides (TMDs) facilitates the emergence of two degenerate yet non-equivalent valleys, making these materials ideal for investigating valleytronic phenomena. Valley polarization can be induced by negating valley degeneracy, enabling selective valley manipulation in logic-oriented valleytronic applications. Although optical pumping allows the achievement of valley polarization in 2D TMDs [10], the rapid carrier recombination severely restricts this technique’s scalability. In order to break the time-reversal symmetry, magnetism can be employed to provide an alternative pathway for valley polarization, but many specific constraints remain. Valley polarization induced by a 1.0 T magnetic field reaches merely 0.1 meV [11], revealing inefficient magnetic manipulation. Magnetic proximity engineering can successfully negate valley degeneracy [12]. However, it introduces a reconfiguration of substantial electronic bands, which suppresses valley properties. Magnetic doping can cause atoms to cluster, so carriers are scattered to degrade valleytronic device performance [13]. 

To circumvent these issues, ferromagnetic (FM) materials with valley properties, known as ferrovalley (FV) materials [14], are more desirable. The first-discovered 2D FV material, 2H-VSe_2_ [14], features an intrinsic FM order, which can break time-reversal symmetry. Also, it was found that monolayer VSe_2_ exhibits spin–valley coupling, allowing regulatable valley polarization via external fields. Subsequently, investigators have reported numerous FV systems, such as ScBrI [15], RuClBr [16], VCGeN_4_ [17], GdI_2_ [18], LaBrI [19], and CeI_2_ [20]. Cheng et al. found that monolayer GdI_2_ is a compelling FV material for valleytronic applications [18]. Sheng et al. reported that FM monolayer CeI_2_ has excellent stability and exhibits large valley polarization [20]. Nevertheless, it is worth noting that some shortcomings remain, including low magnetic transition temperatures, low experimental feasibility, and a low level of valley polarization. These limitations affect the application of FV materials, and the core targets remain high levels of valley polarization, high magnetic transition temperatures, and broad applicability. Designing and discovering ideal materials constitutes a formidable challenge for experimental studies and valleytronic implementations [21,22,23,24].

Recently, monolayer XSi_2_N_4_ (X = Mo, W) was fabricated via the chemical vapor deposition method in an experiment [25]. Moreover, monolayer TiTe_2_ has been obtained via molecular beam epitaxy [26]. Here, inspired by monolayer TiTe_2_, a novel 2D valleytronic material TiAlTe_3_ is proposed. Via first-principles calculations, we report that monolayer TiAlTe_3_ is an excellent FV material with superb structural stability and strong FM coupling. When spin–orbital coupling (SOC) is included and monolayer TiAlTe_3_ is magnetized toward an out-of-plane orientation, a high level of spontaneous valley polarization, amounting to 103 meV, can be observed in the bottom conduction band. Moreover, because of its powerful valley-contrasting Berry curvature, the anomalous valley Hall effect (AVHE) emerges under the influence of an in-plane electric field. Our investigations indicate that monolayer TiAlTe_3_ is a promising material for both experimental research and practical applications. 

## 2. Computational Details

Based on density functional theory, first-principles calculations were carried out by means of the projector-augmented wave method using the Vienna Ab initio Simulation Package [27,28,29]. The exchange-correlation potential was set according to the Perdew–Burke–Ernzerhof (PBE) functional of generalized gradient approximation [30]. Spin polarization was included in all calculations. The cutoff energy was set to 500 eV. A vacuum space of 25 Å in the *c* direction was adopted to avoid interaction between adjacent layers. The total energy and force convergence criteria were set to 10^−6^ eV and 0.01 eV/Å, respectively. The PBE+U method [31] was applied to describe the Ti-3*d* orbitals, and U and J were set to 3 and 0 eV, respectively [32,33]. For calibrating the PBE+U method, the Heyd–Scuseria–Ernzerhof (HSE06) hybrid functional [34] was applied. The Brillouin zone was sampled utilizing a Г-centered 17 × 17 × 1 k mesh for the unit cell and a mesh with dimensions of 17 × 9 × 1 for the rectangle supercell. A 4 × 4 × 1 supercell was used to analyze phonon dispersion using PHONOPY software (the version number: 2.15.1) [35,36]. An Ab initio molecular dynamics (AIMD) [37] simulation was performed over a 4 × 4 × 1 supercell at a temperature of 300 K for 8 ps. Berry curvature was calculated using the WANNIER90 package (the version number: 3.1.0) [38,39].

## 3. Results

**Figure 1:**
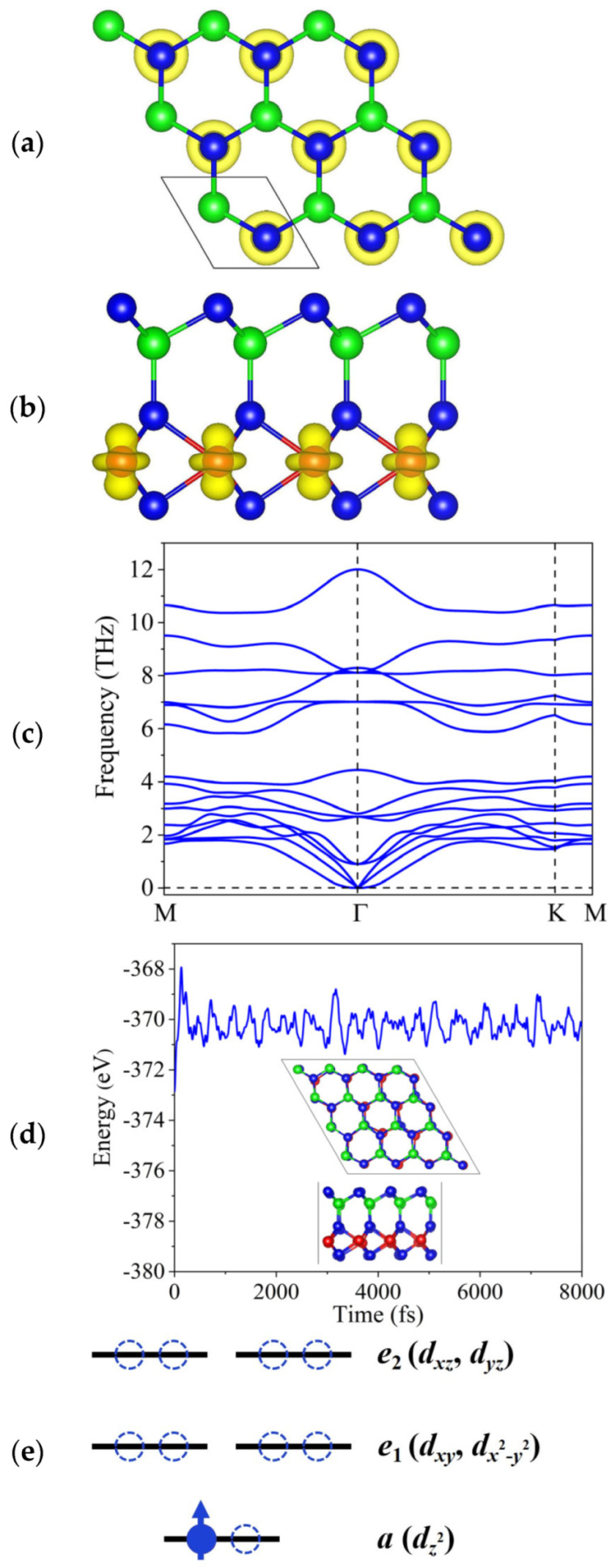
The (**a**) top and (**b**) side views of monolayer TiAlTe_3_ along with the spin charge density. The blue, green, and red balls represent Te, Al, and Ti atoms, respectively. (**c**) The phonon dispersion of monolayer TiAlTe_3_. (**d**) The AIMD of monolayer TiAlTe_3_ at 300 K. (**e**) The splitting of Ti-3*d* orbitals under trigonal prismatic crystal field.

Figure 1a,b show the optimized crystal structures of monolayer TiAlTe_3_ with a hexagonal lattice structure and a *P*3*m*1 space group (No. 156). Each unit cell includes one Ti atom, one Al atom, and three Te atoms, and the stacking sequence is Te-Al-Te-Ti-Te. Clearly, broken space inversion symmetry can be observed in monolayer TiAlTe_3_. The optimized lattice constant *a* of monolayer TiAlTe_3_ is 4.07 Å. The thermal and dynamic stabilities were evaluated through AIMD simulation and phonon dispersion, respectively. According to Figure 1c, the absence of an imaginary frequency shows the dynamical stability of monolayer TiAlTe_3_. The total energy fluctuated near the equilibrium value after 8 ps, and no evident disruption was observed for monolayer TiAlTe_3_ (see Figure 1d), proving that it is thermally stable at room temperature.

The formation energy is calculated as follows:

(1) $$E_{\mathrm{form}}=E_{{\mathrm{TiAlTe}}_{3}}-E_{{\mathrm{TiTe}}_{2}}-\frac{1}{2}E_{{\mathrm{Al}}_{2}{\mathrm{Te}}_{2}}$$

Here, $E_{{\mathrm{TiAlTe}}_{3}}$, $E_{{\mathrm{TiTe}}_{2}}$, and $E_{{\mathrm{Al}}_{2}{\mathrm{Te}}_{2}}$ are the energy of the monolayers TiAlTe_3_, TiTe_2_, and α-AlTe, respectively. The $E_{\mathrm{form}}$ of monolayer TiAlTe_3_ is −1.75 eV, indicating that individual TiTe_2_ and *α*-AlTe systems cannot be obtained through the spontaneous decomposition of monolayer TiAlTe_3_. For the hexagonal structure, two independent elastic constants of *C*_11_ and *C*_12_ were obtained: 52.33 Nm^−1^ and 15.31 Nm^−1^, respectively. These *C_ij_* meet the Born criterion [40,41], namely, *C*_11_ > 0 and *C*_11_ > |*C*_12_|, verifying the structure’s mechanical stability. Naturally, the fabrication scheme for monolayer TiAlTe_3_ is discussed due to its excellent stability. We hypothesize that monolayer TiAlTe_3_ can be synthesized by depositing an *α*-AlTe structure on monolayer TiTe_2_ [26]. 

To investigate the magnetic ground state of monolayer TiAlTe_3_, the total energy difference between antiferromagnetic (AFM) and FM states was calculated by employing one rectangle supercell. Under a U value of 3 eV, the results show that the FM state is energetically lower than the AFM state by 44.6 meV for monolayer TiAlTe_3_. The energy differences between FM and AFM states as a function of U value are plotted in Figure S1. The total magnetic moment per unit cell was determined to be 1 *μ*_B_. The magnetic moment is primarily from the Ti atom (see Figure 1a,b), and thus the contributions of Al and Te atoms are very small, a phenomenon that can be attributed to the special configuration. Each Ti atom is coordinated by six Te atoms, forming one trigonal prismatic. Therefore, according to Figure 1e, the 3*d* orbitals of each Ti atom split into three portions: *e*_1_ (*d_xy_*, *d_x*^2^*_*_−*y*^2^_), *e*_2_ (*d_xz_*, *d_yz_*), and *a* (*d_z*^2^*_*). The electronic configuration of one Ti atom is 3*d*^2^4*s*^2^. In order to form monolayer TiAlTe_3_, each Ti atom contributes three electrons to Te atoms. One unpaired electron occupies the lower-energy *d_z*^2^*_* orbital, which generates a magnetic moment of 1 *μ*_B_ per Ti atom. We can comprehend the FM coupling mechanism of monolayer TiAlTe_3_ by assessing superexchange (SE) and direct-exchange (DE) interactions. The length between the nearest-neighbor Ti atoms (*d*_Ti-Ti_) for monolayer TiAlTe_3_ is 4.07 Å, indicating that AFM DE interactions cannot occur. In addition, based on the Goodenough–Kanamori–Anderson theory [42,43,44], the SE interactions in next-nearest-neighbor Ti atoms via the Te atom (namely, Ti-Te-Ti) also tend to appear FM coupling. As shown in Figure 1b, the bond angles of Ti-Te-Ti for monolayer TiAlTe_3_ were discovered to be 88.5° and 90.6° in the upper and bottom SE routes, respectively, both being close to 90°. Hence, the intrinsic FM coupling of monolayer TiAlTe_3_ is governed by the SE interaction between the Te-5*p* and Ti-3*d* orbitals. We conclude that Ti^3+^-related systems are built by multiple coordinate atoms, which can appear in various phases and exhibit diversity.

On the basis of the Mermin–Wagner theory [45], powerful thermal fluctuations can easily destroy long-range FM ordering. Nevertheless, the 2D ferromagnets Cr_2_Ge_2_Te_6_ [46] and CrI_3_ [47] exhibit magnetic anisotropy governed by magnetic anisotropy energy (MAE), which plays a significant role in realizing long-range FM ordering. The MAE is estimated using the following equation:

(2) $$E_{\mathrm{MAE}}=E_{x}-E_{z}$$

where $E_{x}$ and $E_{z}$ signify the total energy for the magnetization in IP and out-of-plane (OP) orientations, respectively. The MAE calculated was −418 μeV. This confirms that the easy magnetization axis of monolayer TiAlTe_3_ is in the IP orientation, indicating there is no energy obstacle when the magnetization rotates in the *xy* plane. In this situation, monolayer TiAlTe_3_ is a 2D *XY* magnet. The Berezinsky–Kosterlitz–Thouless transition can happen at a critical temperature:

(3) $$T_{C}=1.335J/k_{B}$$

where $k_{B}$ and J are the Boltzmann constant and nearest-neighbor exchange parameter ($J=\left(E_{\mathrm{AFM}}-E_{\mathrm{FM}}\right)/8{\left|S\right|}^{2}$), respectively. The J as a function of U value is shown in Figure S2. With $\left|S\right|=1/2$ and J=22.3 meV in one rectangle supercell, $T_{C}$ was found to be 344 K. The perfect combination of a sizable MAE and a high magnetic transition temperature renders monolayer TiAlTe_3_ a prospective platform for developing spintronic and valleytronic devices.

**Figure 2:**
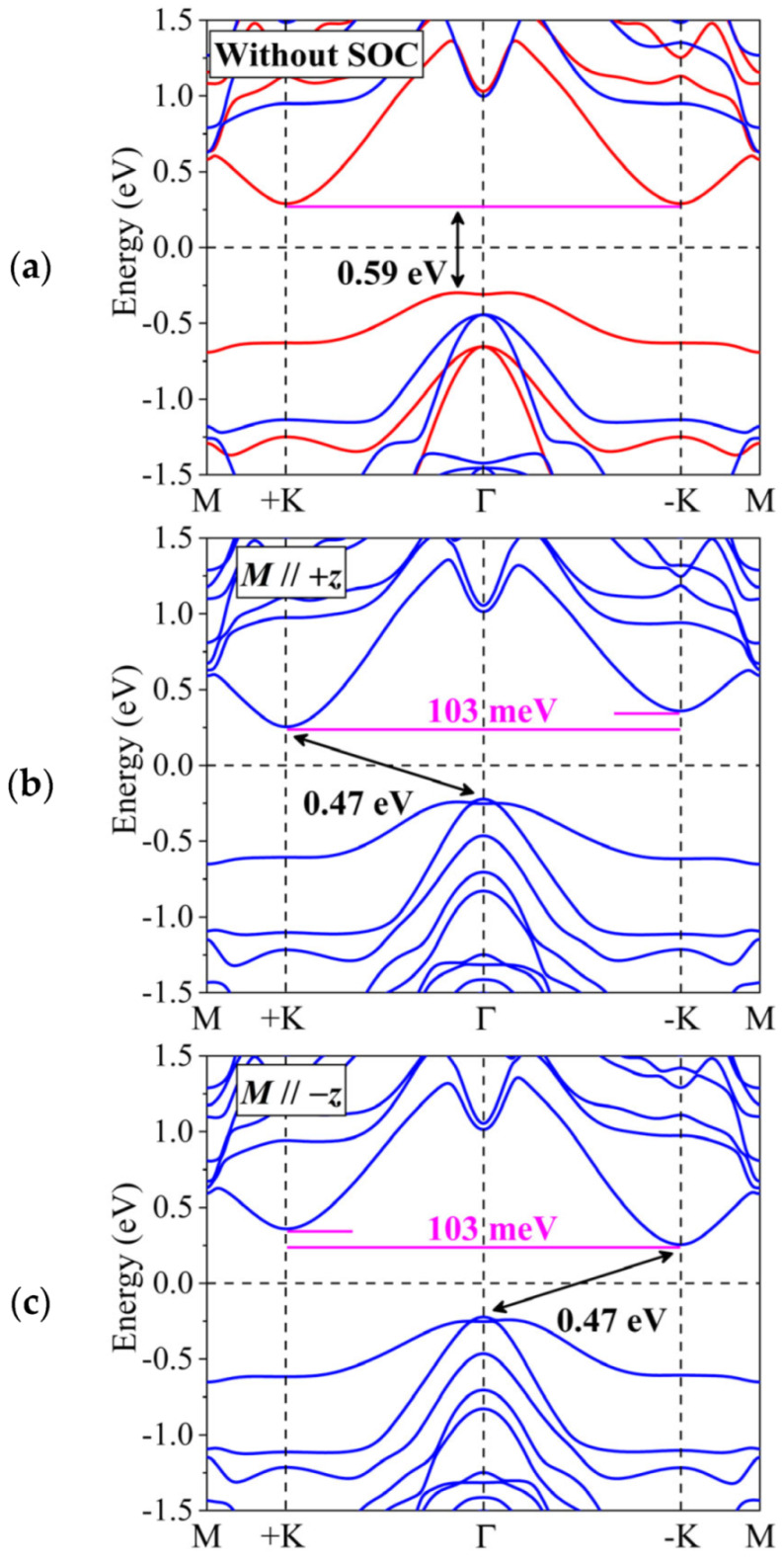
In the FM ground state, the band structures of monolayer TiAlTe_3_ (**a**) without SOC and (**b**,**c**) with SOC for magnetization in (**b**) positive *z* and (**c**) negative *z* directions, respectively. In (**a**), the red and blue lines represent spin-up and spin-down states, respectively.

The electronic properties under different U values are investigated (see Figure S3), and the calculation by the HSE06 functional is employed for comparative analysis (see Figure S4). The band structure of monolayer TiAlTe_3_ without SOC is presented in Figure 2a, exhibiting noticeable spin splitting due to magnetic exchange coupling. Evidently, monolayer TiAlTe_3_ is an indirect semiconductor with a band gap of 0.59 eV. The conduction band minimum (CBM) is located at +K and −K high-symmetry points, while the valence band maximum (VBM) is located between +K (−K) and Г points. The VBM and CBM come from the same spin-up state. We speculate that a rational carrier doping can induce the movement of the Fermi level ($E_{f}$), so the electronic properties of monolayer TiAlTe_3_ allow for the creation of a half-metal, which facilitates the development of advanced nanodevices. Additionally, in the bottom conduction band, a pair of energetically degenerate valleys emerges at the +K and −K points, and monolayer TiAlTe_3_ is a potential FV material. Because the space inversion symmetry is naturally broken, the two degenerate valleys have non-equivalent properties [14]. 

Significantly, the magnetization orientation can be varied from IP to OP under an external magnetic field. According to Figure 2b, monolayer TiAlTe_3_ still shows indirect semiconductor properties, and the band gap is 0.47 eV. The SOC negates the degeneracy between +K and −K valleys in the bottom conduction band, and spontaneous valley polarization equal to 103 meV is induced in monolayer TiAlTe_3_. This corresponds to employing an enormous external magnetic field of about 515–1030 T. The valley polarization of 103 meV is superior to that of numerous other FV systems discovered, such as ScBrI (90 meV) [15], VCGeN_4_ (46 meV) [17], and LaBrI (59 meV) [19]. According to Figure 2c, when monolayer TiAlTe_3_ is magnetized toward a −*z* orientation, the valley polarization and spin are concurrently reversed. The valley states in monolayer TiAlTe_3_ can be regulated by transforming its magnetization orientation.

The related physical mechanism of spontaneous valley polarization stems from the synergistic interplay between SOC and spin-polarized effects. Firstly, when the spin-polarized effect is not involved, the spin degeneracy can be split by the SOC effect at the +K and −K valleys. Due to the protection of time-reversal symmetry, the opposite spin states at the +K and −K valleys can be observed:

(4) $$E_{\mathrm{SO}↑}\left(+K\right)\neq E_{\mathrm{SO}↑}\left(-K\right)$$

(5) $$E_{\mathrm{SO}↑}\left(+K\right)=E_{\mathrm{SO}↓}\left(-K\right)$$

Here, E and ↑ (↓) are the energy of the valley in the bottom conduction band and the spin symbol, respectively. Secondly, as shown in Figure 2a, when the SOC effect is excluded, the identical spin symbol in the +K and −K valleys is forced by spin-polarized effect; that is,

(6) $$E_{\mathrm{SP}↑}\left(+K\right)=E_{\mathrm{SP}↑}\left(-K\right)$$

Therefore, when both SOC and spin-polarized effects are simultaneously included, the +K and −K valleys in monolayer TiAlTe_3_ do not exhibit energetic degeneracy, resulting in spontaneous valley polarization.

By analyzing the orbital contributions to the +K and −K valleys, a quantitative model linking valley polarization with magnetization orientation was derived. Due to the broken time-reversal symmetry, the interplay between opposite spin states was negated. Therefore, the SOC Hamiltonian is written as follows:

(7) $${\hat{H}}_{\mathrm{SOC}}=\lambda_{\mathrm{SOC}}\hat{L}\cdot \hat{S}\approx {\hat{H}}_{\mathrm{SOC}}^{0}=\lambda_{\mathrm{SOC}}{\hat{S}}_{z^{\prime }}\left({\hat{L}}_{z}\cos\theta +\frac{1}{2}{\hat{L}}_{+}e^{-i\varphi }\sin\theta +\frac{1}{2}{\hat{L}}_{-}e^{+i\varphi }\sin\theta \right)$$

**Figure 3:**
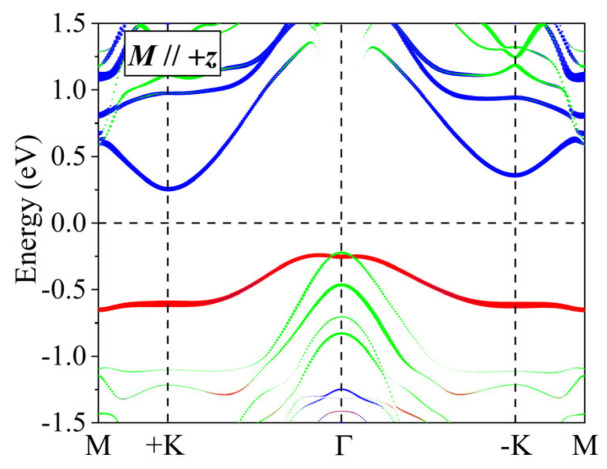
In the FM ground state, the orbital-projected band structure of monolayer TiAlTe_3_ with SOC for magnetization in the positive *z* direction. The red, green, and blue symbols denote the *d_z*^2^*_*, *d_yz_*/*d_xz_*, and *d_x*^2^*_*_−*y*^2^_/*d_xy_* orbital components of a Ti atom, respectively.

Here, $\hat{L}$ is the orbital angular momentum, with the coordinates $\left(x,y,z\right)$, and $\hat{S}$ is the spin angular momentum, with the coordinates $\left(x^{\prime },y^{\prime },z^{\prime }\right)$. The θ and φ are polar angles. As shown in Figure 3, the +K and −K valleys in the bottom conduction band mainly arise from the Ti-3*d_xy_* and Ti-3*d_x*^2^*_*_−*y*^2^_ orbitals, and the group symmetry of monolayer TiAlTe_3_ is *C*_3*v*_. So, the basis functions are expressed as follows:

(8) $$|\phi_{c}^{\tau }\rangle =\left(|3d_{x^{2}-y^{2}}\rangle +i\tau |3d_{xy}\rangle \right)/\sqrt{2}$$

Here, τ=±1 and c are the valley index and conduction band, respectively. Based on $\hat{L}$ and ${\hat{L}}_{z}$, the atomic orbitals can be expressed as follows:

(9) $$|3d_{x^{2}-y^{2}}\rangle =\left(|2,-2\rangle +|2,+2\rangle \right)/\sqrt{2}$$

(10) $$|3d_{xy}\rangle =i\left(|2,-2\rangle -|2,+2\rangle \right)/\sqrt{2}$$

Hence, the energy levels for the +K and −K valleys in the bottom conduction band can be written as follows:

(11) $$E_{c}^{+K}=\langle \psi_{c}^{+}\left|{\hat{H}}_{\mathrm{SOC}}^{0}\right|\psi_{c}^{+}\rangle =\langle 2,-2\left|{\hat{H}}_{\mathrm{SOC}}^{0}\right|2,-2\rangle =-2\lambda_{\mathrm{SOC}}{\hat{S}}_{z^{\prime }}\cos\theta$$

(12) $$E_{c}^{-K}=\langle \psi_{c}^{-}\left|{\hat{H}}_{\mathrm{SOC}}^{0}\right|\psi_{c}^{-}\rangle =\langle 2,+2\left|{\hat{H}}_{\mathrm{SOC}}^{0}\right|2,+2\rangle =+2\lambda_{\mathrm{SOC}}{\hat{S}}_{z^{\prime }}\cos\theta$$

Valley polarization is expressed as follows:

(13) $$\Delta_{c}=E_{c}^{-K}-E_{c}^{+K}=4\lambda_{\mathrm{SOC}}{\hat{S}}_{z^{\prime }}\cos\theta =4\alpha_{\mathrm{SOC}}\cos\theta$$

Here, $\alpha_{\mathrm{SOC}}$ is the constant associated with SOC. The trend of valley polarization derived from the model is in agreement with that from first-principles calculations, as illustrated in Figure 2b.

After the spontaneous valley polarization of monolayer TiAlTe_3_ was identified, the Berry curvature was explored to determine electronic transport properties. We calculated the Berry curvature via the Kudo formula [48]:

(14) Ωzk=−∑n∑n≠n′fn2Imψnkυ^xψn′kψn′kυ^yψnkEn−En′2

**Figure 4:**
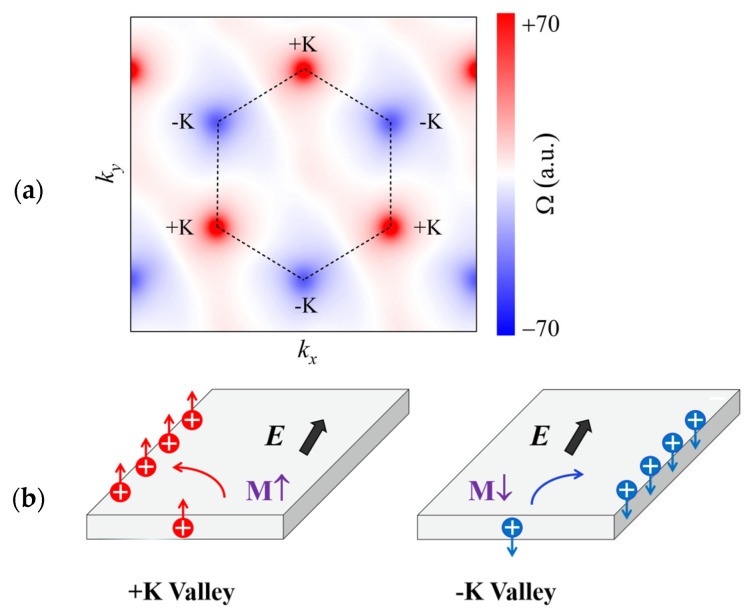
The Berry curvature of monolayer TiAlTe_3_ in the FM ground state when the magnetization orientation is OP (**a**). (**b**) Schematic diagram of AVHE for hole-doped monolayer TiAlTe_3_.

Here, $f_{n}$ is the Fermi–Dirac distribution function, and $\langle \psi_{nk}|$ is the Bloch wave function with an eigenvalue of $E_{n}$. ${\hat{\upsilon }}_{x}$ and ${\hat{\upsilon }}_{y}$ are velocity operators in the x and y directions, respectively. The calculated Berry curvature of monolayer TiAlTe_3_ is plotted in Figure 4a. Apparently, the Berry curvature reveals opposite signs and identical absolute values in the neighborhood of the +K and −K valleys, which indicates the valley-contrasting property of monolayer TiAlTe_3_. 

The Bloch electrons can obtain an anomalous velocity, υ∝E×Ω, when an IP electric field is applied [49]. The carriers can shift to opposite boundaries owing to powerful valley-contrasting Berry curvature. The immense spontaneous valley polarization is realized in monolayer TiAlTe_3_, so the $E_{f}$ can be moved between the +K and −K valleys in the bottom conduction band. The observed instances of hole shift in *p*-type monolayer TiAlTe_3_ under an IP electric field are displayed in Figure 4b. For magnetization in the +*z* direction, as illustrated in Figure 4b, the spin-up holes from the +K valley can transversely shift to the left boundary. For magnetization in the −*z* direction, the spin-down holes from the −K valley move to the right boundary, according to Figure 4b. As a consequence, AVHE is demonstrated in monolayer TiAlTe_3_. Furthermore, accompanied by the AVHE, extra-spin Hall current and charge can occur, because carriers with the same spin and charge are accumulated on the boundary of the material. The remarkable synergistic combination of valley Hall current, charge, and spin provides a promising pathway for integrating electronics, spintronics, and valleytronics.

## 4. Conclusions

In conclusion, monolayer TiAlTe_3_ has theoretically been identified as an FM material with excellent structural stability and a high magnetic transition temperature. Spontaneous valley polarization of 103 meV could be observed when the magnetization orientation is OP. The valley states in monolayer TiAlTe_3_ could be modulated by transforming its magnetization orientation. Via the doping of the carriers, the AVHE was realized in monolayer TiAlTe_3_. Our research demonstrates that monolayer TiAlTe_3_ exhibits spin-valley coupling, offering a potential platform for spintronic and valleytronic explorations. 

## Data Availability

The original contributions presented in this study are included in the article. Further inquiries can be directed to the corresponding authors.

## Supplementary Materials

The following supporting information can be downloaded at https://www.mdpi.com/article/10.3390/ma18102396/s1. Figure S1. The energy differences ($\Delta E=E_{\mathrm{AFM}}-E_{\mathrm{FM}}$) between FM and AFM states as a function of U value. Figure S2. The nearest-neighbor exchange parameter J of monolayer TiAlTe_3_ as a function of U value. Figure S3. Under the FM ground state, the band structures of monolayer TiAlTe3 calculated from PBE+U method without SOC. The U values are chosen as (a) 0 eV, (b) 1 eV, (c) 2 eV, and (d) 4 eV. Figure S4. Under the FM ground state, the band structure of monolayer TiAlTe_3_ by the HSE06 functional without SOC.
