# Supplementary material for: Monolayer TiAlTe3: A Perfect Room-Temperature Valleytronic Semiconductor
Source: Materials (Basel). 2025 May 21;18(10):2396. doi: 10.3390/ma18102396 (PMC12113017; doi:10.3390/ma18102396)
Supplement: Supplementary file 1 [file materials-18-02396-s001.zip › materials-3616662-supplementary.pdf]

*Supplementary*

# Monolayer $TiAlTe_3$: A perfect room-temperature valleytronic semiconductor

**Kang Jia [1,2], Chang-Wen Zhang [1], Zi-Ran Wang [3,4,*] and Pei-Ji Wang [1,*]**

[1] School of Physics and Technology, Institute of Spintronics, University of Jinan, Jinan 250022, China
[2] School of Physics and Physical Engineering, Qufu Normal University, Qufu 273165, China
[3] Key Laboratory of High-Effciency and Clean Mechanical Manufacture of MOE, School of Mechanical Engineering, Shandong University, Jinan 250061, China
[4] Suzhou Research Institute, Shandong University, Suzhou 215009, China
* Correspondence: wangziran@sdu.edu.cn (Z.-R.W.); ss_wangpj@ujn.edu.cn (P.-J.W.)

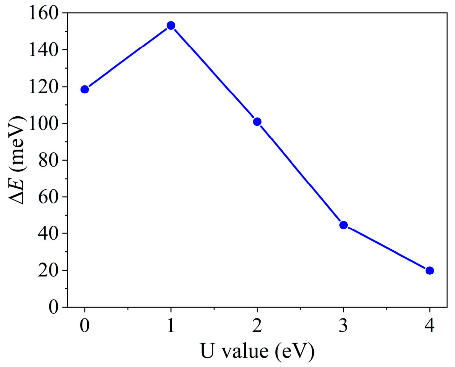


**Figure S1.** The energy differences ($\Delta E = E_{\mathrm{AFM}} - E_{\mathrm{FM}}$) between FM and AFM states as a function of U value.

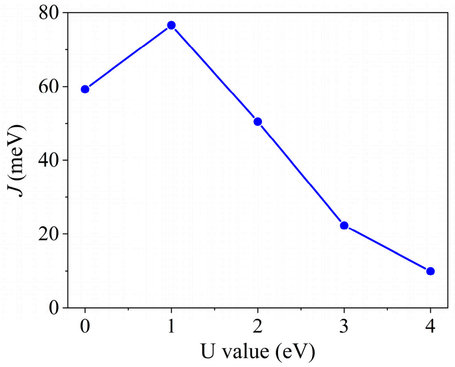


**Figure S2.** The nearest-neighbor exchange parameter $J$ of monolayer $TiAlTe_3$ as a function of U value.

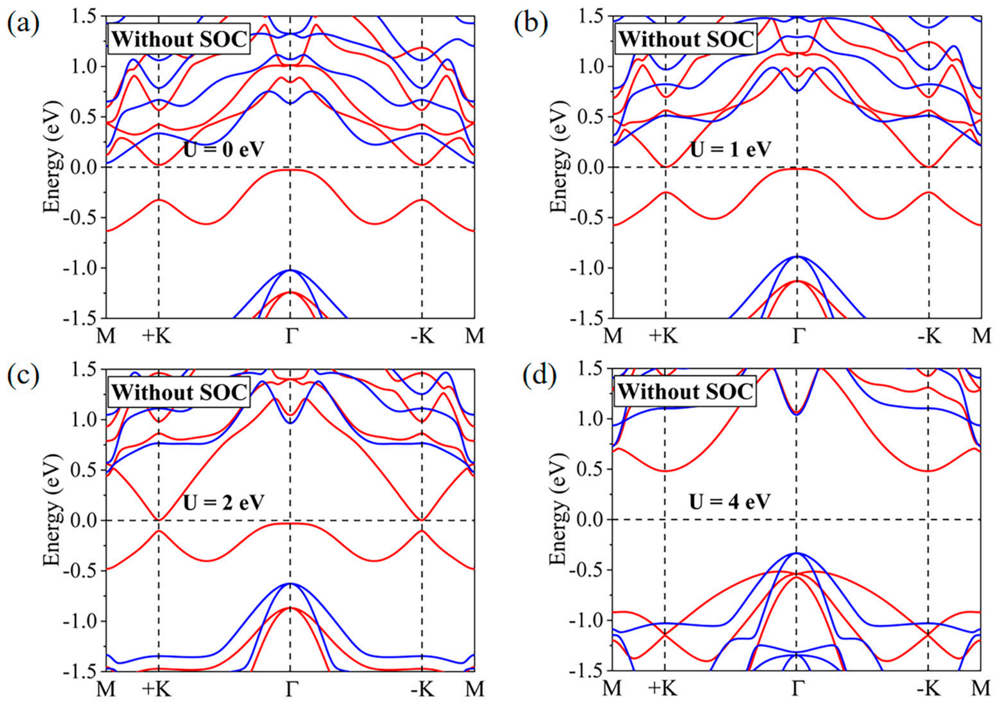


**Figure S3.** Under the FM ground state, the band structures of monolayer $TiAlTe_3$ calculated from PBE+U method without SOC. The U values are chosen as (a) 0 eV, (b) 1 eV, (c) 2 eV, and (d) 4 eV.

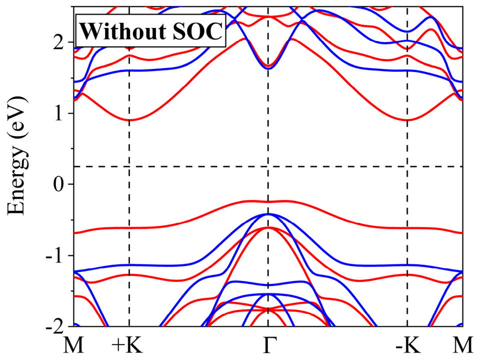


**Figure S4.** Under the FM ground state, the band structure of monolayer $TiAlTe_3$ by the HSE06 functional without SOC.

The nearest-neighbor exchange parameter $J$ is determined by the energy difference between the FM and AFM states. The energies of AFM and FM states can be calculated from the following equations

$E_{\mathrm{FM}} = E_0 - (6J + 2A)S^2$ (S1)

$E_{\mathrm{AFM}} = E_0 + (2J - 2A)S^2$ (S2)

Here, $E_{\mathrm{FM}}$ and $E_{\mathrm{AFM}}$ are the energies of FM and AFM states, respectively. $E_0$ and $A$ are the total energy of system and anisotropy parameter, respectively. So, the nearest-neighbor exchange parameter $J$ can be calculated by

$J = (E_{\mathrm{AFM}} - E_{\mathrm{FM}}) / 8|S|^2$ (S3)

As shown in Figure S1 and Figure S2, with increasing U value, the $\Delta E$ and $J$ first increase, and then decrease.

The effect of different U values on electronic properties of monolayer $TiAlTe_3$ is investigated to acquire the accurate electronic structures. According to Figure S3 and Figure 2(a), monolayer $TiAlTe_3$ has semiconductor properties for these U values (U = 0, 1, 2, 3, 4 eV). In particular, when the U value is set to 3 eV (see Figure 2(a)), the band structure is very consistent with that calculated from HSE06 functional (see Figure S4). It is observed that the band gap of 1.14 eV by the HSE06 functional is larger than that by the PBE+U method. The U = 3 eV is utilized in the following discussion.
